# Supplementary material for: Early detection of esophageal cancer: Evaluating AI algorithms with multi-institutional narrowband and white-light imaging data
Source: PLoS One. 2025 Apr 4;20(4):e0321092. doi: 10.1371/journal.pone.0321092 (PMC11970661; doi:10.1371/journal.pone.0321092)
Supplement: Attachment — Submitted filename: rivision_0224.docx [file pone.0321092.s003.docx]

**Journal Requirements**

1. Please review your reference list to ensure that it is complete and correct. If you have cited papers that have been retracted, please include the rationale for doing so in the manuscript text, or remove these references and replace them with relevant current references. Any changes to the reference list should be mentioned in the rebuttal letter that accompanies your revised manuscript. If you need to cite a retracted article, indicate the article’s retracted status in the References list and also include a citation and full reference for the retraction notice.

**Response**

It is important to write references completely and accurately as required by the journal. We, the authors, fully agree with the journal's requirements, as the references are what support the research in the paper. We have checked and reviewed them thoroughly. We have reviewed the references of the papers and none of them have been retracted. We appreciate your comments regarding the journal to improve the quality of the paper.

**Response to Reviewer 1**

The proposed approach and the result will be helpful for many researchers. The paper has been carefully revised by the authors. After all revisions, the quality of the paper has been improved. Therefore, the revised version can be accepted for publication.

**Response**

Thank you for your positive comments on this paper. I think the quality of the paper has improved a lot of thanks to the reviewer's comments. Thank you for your detailed comments on improving the paper.

**Response to Reviewer 2**

1. I only saw the TP, TN, FP, and FN but not the confusion matrices that were used to calculate them. It would be better if those matrices were shown.

**Response**

We appreciate the reviewer’s suggestion regarding the confusion matrix. However, in object detection tasks, the concept of True Negative (TN) is not well-defined, as it is in classification tasks. Detection models focus on identifying objects (lesions) within an image, and their performance is typically evaluated using Precision, Sensitivity, FPPI (False Positives Per Image), and mAP (Mean Average Precision) rather than a traditional confusion matrix. In our study, we have already provided a detailed table summarizing TP, FP, and FN values to clearly present the detection performance of our models. Metrics such as precision and sensitivity provide a more detailed understanding of a model's detection capabilities, and FPPI helps to assess a model's tendency to generate false positives in a detection setting. However, we appreciate your suggestion, and in future work we will consider incorporating the confusion matrix if it provides more insight into model performance. You raised this important point, which has helped us to reflect on our evaluation approach.

2. It would have been good if there were extra novelties highlighted rather than only dataset and hyperparameter tuning.

**Response**

We appreciate the reviewer’s comment regarding the novelty of our study. Beyond dataset expansion and hyperparameter tuning, our work provides significant contributions: (1) multi-institutional dataset validation, improving model generalization and addressing dataset bias; (2) comparative analysis of YOLOv5 and RetinaNet, offering practical insights for clinical applications; (3) integration of both WLI and NBI imaging, enabling a more comprehensive assessment of esophageal cancer detection; (4) detailed analysis of false positives and false negatives, enhancing interpretability and clinical relevance; and (5) proposed future enhancements using synthetic data generation, suggesting broader applications beyond endoscopy. We have clarified these contributions in the revised manuscript. We sincerely appreciate the reviewers' feedback, which helped us to further clarify the novelty and contribution of our work.
