# Supplementary material for: Early detection of esophageal cancer: Evaluating AI algorithms with multi-institutional narrowband and white-light imaging data
Source: PLoS One. 2025 Apr 4;20(4):e0321092. doi: 10.1371/journal.pone.0321092 (PMC11970661; doi:10.1371/journal.pone.0321092)
Supplement: Attachment — Submitted filename: rivision_0113.docx [file pone.0321092.s002.docx]

**Editor**

1. Please ensure that your manuscript meets PLOS ONE's style requirements, including those for file naming. The PLOS ONE style templates can be found at

https://journals.plos.org/plosone/s/file?id=wjVg/PLOSOne_formatting_sample_main_body.pdf and

<https://journals.plos.org/plosone/s/file?id=ba62/PLOSOne_formatting_sample_title_authors_affiliations.pdf>

**Response**

As the editor mentioned, it appears that the format of this paper did not fit the requirements defined by PLOS ONE. Therefore, the authors revised and supplemented the overall format of the paper by referring to the PDF files mentioned by the editor in the email. Thanks to the editor's comments, the completeness of the paper has improved. Thank you.

2. Please note that PLOS ONE has specific guidelines on code sharing for submissions in which author-generated code underpins the findings in the manuscript. In these cases, we expect all author-generated code to be made available without restrictions upon publication of the work. Please review our guidelines at https://journals.plos.org/plosone/s/materials-and-software-sharing#loc-sharing-code and ensure that your code is shared in a way that follows best practice and facilitates reproducibility and reuse.

**Response**

We appreciate the reviewer's comments about code sharing. In our research, we utilized the publicly available codebases of YOLOv5 and RetinaNet as the basis for our work. The implementations can be accessed from the following GitHub repositories. YOLOv5 (https://github.com/ultralytics/yolov5) and RetinaNet (https://github.com/fizyr/keras-retinanet). These repositories are publicly accessible and adhere to best practices for reproducibility and reuse. Although our work is primarily concerned with the adaptation and fine-tuning of these existing frameworks for esophageal lesion detection, we have ensured that all modifications and hyperparameter settings are described in detail in the manuscript. This allows readers and researchers to effectively reproduce our results using the reference repositories.

3. Thank you for stating the following financial disclosure:

[This work was supported by a grant from the Korean Gastrointestinal Endoscopy Research Foundation (2021 Investigation Grant), and by the Gachon University Gil Medical Center (Grant number: FRD2022-12), and by the Gachon University research fund of 2023(GCU-202308020001)].

Please state what role the funders took in the study. If the funders had no role, please state: "The funders had no role in study design, data collection and analysis, decision to publish, or preparation of the manuscript."

If this statement is not correct you must amend it as needed.

Please include this amended Role of Funder statement in your cover letter; we will change the online submission form on your behalf.

**Response**

Thank you for providing funding and for carefully reviewing and confirming the details of the paper. The funders who provided funding for this paper had no role in the preparation of the manuscript. A description of the role of the funder was included in the cover letter. Please confirm.

**Response to Reviewer 1**

We sincerely thank the reviewer for their constructive feedback, which has significantly improved the quality of our manuscript. Below, we address each comment in detail:

1. In the abstract section, some important numerical results should be added to inform the readers about the performance and effectiveness of the proposed method.

**Response**

As per the reviewer's comments, we have added some important numerical results in the Abstract section to inform the reader about the performance and effectiveness of the proposed method. We have revised the abstract to include key numerical results that demonstrate the effectiveness of the proposed method. Specifically, we state that RetinaNet achieved 98.4% precision and 91.3% sensitivity on the NBI dataset and YOLOv5 achieved 93.7% precision and 89.9% sensitivity on the WLI dataset. This content has also been added to the paper. Thank you for mentioning these important points to improve the paper.

Modified and supplemented contents - Page 2, line 7~14

2. Grammatical mistakes should be corrected.

**Response**

We have thoroughly reviewed the manuscript and addressed all grammatical issues. The language throughout the manuscript has been refined to enhance clarity and readability. For example, ambiguous sentences in the abstract and discussion sections have been restructured for better coherence. This content has also been added to the paper. Thank you for leaving a comment to improve the completeness of this paper.

3. The loss and activation functions used in this work should be written.

**Response**

In the revised manuscript, we described the loss and activation functions in more detail. Specifically, we used Focal Loss for the RetinaNet model to address class imbalance and ComputeLoss loss for YOLOv5 to compute the final loss by integrating class loss, objectivity loss, and bounding box loss. In addition, we used ReLU activation function in the backbone layer and Sigmoid activation function in the final layer for classification. This content has also been added to the paper. Thank you for mentioning important points to improve the completeness of this paper.

Modified and supplemented contents - Page 8, line 21~22, Page 9, line 1~2, line 3~4

4. The last section should be "Conclusion". In the discussion section, the advantages/superiority of the proposed approach should be explained.
Also, limitations/drawbacks should be added. Possible extensions of this work and future works should be added.
In future work, the effectiveness of the proposed approach can be investigated for lesion detection from dermoscopy images, which are colored images, and an effective AI-based method is needed in this field although there are recent advancements.

**Response**

Based on the reviewer’s comments, the final section of the manuscript has been renamed ‘Conclusions’ to better reflect its content, and the Discussion section has been expanded and integrated with the Results section under the title ‘Results and Discussion’. This revised section highlights the advantages of the proposed method, including its strong generalization capabilities across multicenter datasets, its high precision and sensitivity for both white-light imaging (WLI) and narrow-band imaging (NBI) data, and its significant contribution to the early diagnosis of esophageal cancer. However, the limitations of the study are also addressed, particularly the potential decline in model performance when encountering unseen datasets with novel artifacts or rare tumor types. These challenges are acknowledged, and potential strategies for overcoming them are discussed. Furthermore, the manuscript has been enhanced to outline future research directions, including extending the proposed approach to applications such as dermatological disease detection and abdominal organ segmentation. These extensions are expected to demonstrate the versatility and effectiveness of the method in addressing challenges in other medical imaging domains. This content has also been added to the paper. Thank you for improving the completeness of this paper by leaving a comment regarding the shortcomings of this paper.

Modified and supplemented contents – Page 11, line 1, Page 16, line 1, line 17~22, Page 17, 6~9, 16~24, Page 18, line 1~2

5. Also, as another future work, the proposed method can be modified to achieve abdominal organ segmentation, such as the liver and kidneys, from grayscale medical images because noise and low contrasts make their segmentations difficult, and atlas or level set-based methods are not always effective.

Therefore, the following statements should be added;
"As a future work, the performance of the proposed method can be tested for the classification of dermatological diseases from dermoscopy images because detection of skin lesions is challenging and an AI-based effective method is still desired in this field despite some recent approaches [R1-R3].
Also, as another future work, the proposed method can be modified to achieve abdominal organ segmentation, such as the liver and kidneys, from grayscale medical images because noise and low contrasts make their segmentations difficult, and atlas or level set-based methods [R4-R9] are not always effective.
R1: Automated Skin Cancer Detection: Where We Are and The Way to The Future, <https://doi.org/10.1109/TSP52935.2021.9522605>
R2: Convolutional neural network based desktop applications to classify dermatological diseases, <https://doi.org/10.1109/IPAS50080.2020.9334956>
R3: Comparative evaluations of cnn based networks for skin lesion classification, Int.Conf.onon Computer Graphics, Visualization, Computer Vision and Image Processing (CGVCVIP)
R4: A comparative performance evaluation of various approaches for liver segmentation from SPIR images, [https://doi.org/10.3906/elk-1304-36](https://doi.org/10.3906/elk-1304-36)
R5: A neural network based kidney segmentation from MR images, <https://doi.org/10.1109/ICMLA.2015.229>
R6: A method for liver segmentation in perfusion MR images using probabilistic atlases and viscous reconstruction, [https://doi.org/10.1007/s10044-017-0666-z](https://doi.org/10.1007/s10044-017-0666-z)
R7: A comparative evaluation for liver segmentation from spir images and a novel level set method using signed pressure force function, Phd thesis,  Izmir Institute of Technology
R8: An automatic level set based liver segmentation from MRI data sets, <https://doi.org/10.1109/IPTA.2012.6469551>
R9: Automatic kidney segmentation using Gaussian mixture model on MRI sequences, [https://doi.org/10.1007/978-3-642-21747-0_4](https://doi.org/10.1007/978-3-642-21747-0_4)

**Response**

Based on the reviewer's comments, we have incorporated the following suggested future research directions into the paper. we have included a future work statement proposing the evaluation of the method's performance for the classification of dermatological diseases using dermoscopy images. We agree that skin lesion detection remains a challenging task, and despite recent advancements, there is still a strong need for an effective AI-based approach in this field. This addition underscores the potential of our method in addressing challenges in other medical imaging domains. we have added a statement suggesting modifications to the proposed method for abdominal organ segmentation, such as the liver and kidneys, from grayscale medical images. We acknowledge that segmentation in such images is often hindered by noise and low contrast, where atlas-based or level-set methods may not be effective. This extension further highlights the adaptability and versatility of the proposed approach. Thank you for improving the completeness of this paper by leaving a comment regarding the shortcomings of this paper.

Modified and supplemented contents – Page 17, 9~15

[31]	Goceri, E. Automated Skin Cancer Detection: Where We Are and The Way to The Future. *In 2021 44th international conference on telecommunications and signal processing (TSP)*, (2021) pp. 48-51. https://doi.org/10.1109/TSP52935.2021.9522605.

[32]	Göçeri, E. Convolutional neural network based desktop applications to classify dermatological diseases. *n 2020 IEEE 4th international conference on image processing, applications and systems (IPAS)*, (2020) pp. 138-143. https://doi.org/10.1109/IPAS50080.2020.9334956.

[33]	Goceri, E., & Karakas, A. A. Comparative evaluations of cnn based networks for skin lesion classification. *In 14th International Conference on Computer Graphics, Visualization, Computer Vision and Image Processing (CGVCVIP), Zagreb, Croatia*, (2020) pp. 1-6.

[34]	Göçeri, E., Ünlü, M. Z., & Dicle, O. A comparative performance evaluation of various approaches for liver segmentation from SPIR images. *Turkish Journal of Electrical Engineering and Computer Sciences*, **23** (2015) 741-768. https://doi.org/10.3906/elk-1304-36.

[35]	Goceri, N., & Goceri, E. A neural network based kidney segmentation from MR images. *In 2015 IEEE 14th international conference on machine learning and applications (ICMLA)*, (2015) pp. 1195-1198. https://doi.org/10.1109/ICMLA.2015.229.

[36]	Dura, E., Domingo, J., Göçeri, E., & Martí-Bonmatí, L. A method for liver segmentation in perfusion MR images using probabilistic atlases and viscous reconstruction. *Pattern Analysis and Applications*, **21** (2018) 1083-1095. https://doi.org/10.1007/s10044-017-0666-z.

[37]	Göçeri, E. A comparative evaluation for liver segmentation from spir images and a novel level set method using signed pressure force function. *Izmir Institute of Technology (Turkey)*, (2013).

[38]	Goceri, E., Unlu, M. Z., Guzelis, C., & Dicle, O. An automatic level set based liver segmentation from MRI data sets. *In 2012 3rd International conference on image processing theory, tools and applications (IPTA)*, (2012) pp. 192-197. https://doi.org/10.1109/IPTA.2012.6469551.

[39]	Goceri, E. Automatic kidney segmentation using Gaussian mixture model on MRI sequences. *In Electrical Power Systems and Computers: Selected Papers from the 2011 International Conference on Electric and Electronics (EEIC 2011) in Nanchang*, **3** (2011) pp. 23-29. https://doi.org/10.1007/978-3-642-21747-0_4.

**Response to Reviewer 2**

We appreciate the reviewer’s insightful comments and constructive feedback, which have helped improve the quality of our manuscript. Below, we provide detailed responses to each comment.

1. Check the typographical error in the last sentence before the conclusion of the introduction. You wrote "om" instead of "on." Then, go through the entire work to rectify typographical errors.

**Response**

Thank you for identifying the typographical error. The word "om" has been corrected to "on" in the introduction. Additionally, we conducted a thorough review of the manuscript and corrected other typographical errors throughout the document to ensure clarity and precision.

Modified and supplemented contents – Page 4, line 24

2. The part of the introduction containing the literature is scanty and must be elaborated on. There is a lot of work on esophageal cancer using AI, and I think you need to reference or add more of this coupled with the evaluation metric values attained.

**Response**

In response to the reviewer's comments, we have revised the relevant research section of the introduction to this manuscript as follows. We have expanded the Introduction to include a comprehensive review of related studies using AI to detect esophageal cancer, highlighting the methods, datasets, and evaluation metrics. Wang et al. developed and validated a deep learning algorithm to evaluate the difference in performance of polyp and adenoma detection using colonoscopy, achieving a sensitivity of 94.3% and specificity of 95.9% [18]. Similarly, Xu et al. designed an architecture for real-time classification and detection of gastric polyps through gastroscopy, achieving 100% sensitivity and 95.4% specificity, with excellent performance in detecting small polyps [19]. For esophageal cancer, Goda et al. showed that magnified endoscopy with narrow-band imaging had a sensitivity of 78% and specificity of 95%, comparable to non-magnified high-resolution endoscopy (sensitivity 72%, specificity 92%) and high-frequency endoscopic ultrasound (sensitivity 83%, specificity 89%), and predicted the depth of invasion of superficial esophageal squamous cell carcinoma, reducing the risk of overestimation by 25% compared to other techniques [20]. Nakagawa et al. found that an AI system using a single-shot multi-detector architecture to assess superficial squamous cell carcinoma achieved a sensitivity of 90.1%, specificity of 95.8%, and accuracy of 91%, which was similar to that of an experienced endoscopist, who achieved a sensitivity of 89.8, specificity of 88.3%, and accuracy of 89.6% [21]. Although there have been many CNN-based studies on lesion detection and diagnosis in various organs, medical data for esophageal squamous cell carcinoma is still limited compared to other datasets, which has led to problems such as overfitting and poor performance on new lesion images [22]. Wang et al. reported that Linked Color Imaging had a specificity of 92.4% and sensitivity of 83.7% for esophageal squamous cell carcinoma screening, which was similar to Lugol Chromoendoscopy with a specificity of 87% and sensitivity of 90.7%, and was promising for screening for squamous cell carcinoma and precancerous lesions in the general population with a much shorter procedure time [23].

Modified and supplemented contents – Page 3, line 25, Page 4, line 1~20

3. It would like you to show the confusion matrices that you used to calculate TP, TN, FP, FN, precision, Sensitivity etc. in the tables and in the text.

**Response**

We have made additions to Tables 2 and 3 of the manuscript based on the reviewer's comments. We have included confusion matrices for both the YOLOv5 and RetinaNet models in the Results section. Thank you for improving the completeness of this paper by leaving a comment regarding the shortcomings of this paper.

Modified and supplemented contents – Page 12, Table 2, Page 14, Table 3

4. If this dataset has been used by any AI algorithm before, a comparative analysis with the authors' results and the existing models' results will help readers and researchers appreciate your efforts.

**Response**

Thank you for your valuable suggestion for a comparative analysis of our dataset and existing AI models. As per the reviewer's comments, we have carefully reviewed the use of our dataset in previous studies and confirmed that it has not been used by other AI algorithms to date. However, we have included a discussion comparing the performance of our model with similar studies utilizing esophageal endoscopy images. This comparison highlights the strengths of our model and places our work in a broader research context. In addition, as a future direction, we plan to further validate and extend the generalization of our model by applying our methodology to other datasets or imaging modalities, which will allow for broader applicability and comparative studies with other datasets in the future. Thank you again for pointing out this important aspect. It has helped us to improve the manuscript.

5. It would have been good if the Accuracy evaluation metric was also used to assess the models.

**Response**

Thank you for sharing your insightful comments about using accuracy as an evaluation metric. Accuracy is commonly used in many classification tasks, but for object detection models such as ours, evaluation metrics such as precision, sensitivity, and false positives per image (FPPI) are often prioritized. These metrics are particularly important in detection problems where the goal is not only to classify objects within an image, but to accurately locate them. In detection tasks, datasets often exhibit class imbalance (e.g., fewer positive samples compared to negative samples), which can make accuracy less informative. Metrics such as precision and sensitivity provide a more detailed understanding of a model's detection capabilities, and FPPI helps to assess a model's tendency to generate false positives in a detection setting. However, we appreciate your suggestion, and in future work we will consider incorporating accuracy if it provides more insight into model performance. You raise this important point, which has helped us to reflect on our evaluation approach.

6. You indicated you were proposing an AI algorithm, but I saw the use of existing models and did not see your novelty in this study. Therefore, I would like you to highlight your novelty.

**Response**

Thank you for your valuable comments on the novelty of the study. We have revised the manuscript to better highlight the unique contribution of the proposed method. Our study demonstrates significant novelty by utilizing a multi-center dataset that combines white light imaging (WLI) and narrowband imaging (NBI) data for esophageal cancer detection, ensuring strong generalizability across different clinical settings. Furthermore, we improved the sensitivity and precision of both imaging modalities by leveraging YOLOv5 and RetinaNet models specifically tailored with hyperparameter optimization designed for esophageal lesion detection. Furthermore, we focused on real-time clinical applicability by carefully balancing model complexity and inference speed, making our approach practical for use in endoscopy. These aspects, which are detailed in the Introduction and Discussion sections, highlight the strengths of our work and its ability to address key challenges in esophageal cancer detection. We sincerely appreciate your feedback, which has helped us to clarify the novelty and contribution of our work.

7. If possible, let's have a link to the dataset you used in this study.

**Response**

As the reviewer comments, sharing the data used in a study enhances the potential for further research and future advances. We are fully aware of this and believe it is important, however, it is not possible for us to publish the patients‘ data in a public repository at Gachon University Gil Hospital, where the patients’ data were provided. If you contact the corresponding author individually, the corresponding author may be able to share the data privately with institutional approval. We thank the reviewers for their generous understanding.

**Response to Reviewer 3**

We appreciate the thoughtful feedback and recommendations provided by Reviewer 3. Our responses to each comment are detailed below.

1. Can the authors provide a clear summary of the key contributions of their work? Highlighting the novelty and practical implications will help better understand the overall significance of the study.

**Response**

We appreciate your insightful comments regarding the novelty of our study and have revised the manuscript to emphasize the unique contributions of our proposed method. This study introduces notable innovations by employing a multicenter dataset that integrates white-light imaging (WLI) and narrow-band imaging (NBI) data for esophageal cancer detection, thereby ensuring robust generalizability across diverse clinical environments. Additionally, we utilized YOLOv5 and RetinaNet models, optimized with hyperparameters specifically designed for esophageal lesion detection, to enhance sensitivity and precision across both imaging modalities. Furthermore, we prioritized real-time clinical applicability by carefully balancing model complexity and inference speed, making our approach suitable for practical implementation in endoscopic procedures. These elements, elaborated in the Introduction and Discussion sections, underscore the strengths of our study and its capacity to address critical challenges in esophageal cancer detection. We are grateful for your feedback, which has allowed us to better articulate the novelty and contributions of this work.

2. Dataset
2-1. Annotation Process: How were the annotations verified? Were domain experts involved in the process?
**Response**

Thank you for pointing out this important aspect. We acknowledge that the annotation process was not described in sufficient detail in the original manuscript. In this study, the regions of interest (ROIs) were annotated by a gastroenterologist with more than 10 years of clinical experience to ensure accuracy and reliability. The annotated ROIs included esophageal cancer lesion areas, and the annotation process was performed using ImageJ, a commercial tool widely used in medical imaging studies. We have incorporated a detailed description of this annotation process into the revised manuscript to clarify our methodology and address your concerns.

Modified and supplemented contents – Page 7, line 3~4

2-2. Data Leakage: Patient-level leakage is a critical concern in medical imaging datasets. Please detail the measures taken to prevent such leakage. For instance, were all images from a single patient assigned exclusively to either the training, validation, or test sets?
**Response**

Thank you for raising this important concern about patient-level data breaches. This is important to ensure the validity and robustness of medical imaging studies. In our study, we took careful steps to prevent patient-level leakage by ensuring that all images from a single patient were assigned to only one of the training, validation, or test sets. This approach ensures that images from the same patient do not appear in more than one dataset, eliminating the risk of duplication and ensuring that the model is evaluated on patient data that is never seen. We also used patient IDs to group images during dataset preparation and maintained separation between datasets. This methodology ensures that the reported performance metrics accurately reflect the generalizability of the model to new unseen patients. We have included this clarification in the revised manuscript to address this concern and clarify the robustness of the experimental setup. We sincerely appreciate the reviewer's comments.

Modified and supplemented contents – Page 6, line 10~13

2-3. Dataset Size: Given the relatively small size of the dataset, were data augmentation techniques employed to increase its size? If yes, please describe the augmentation strategies used.
**Response**

Thank you for sharing the reviewer's comments about data augmentation. We did not use data augmentation techniques in this study. This decision was based on the quality and nature of the dataset, which consisted of real-world clinical images that were carefully curated to retain their original diagnostic value. Given the critical nature of esophageal lesion detection, maintaining the integrity of the original image data was prioritized to ensure the clinical applicability and reliability of the results. As an alternative to data augmentation, we addressed the relatively small dataset size by using a strict train-validate-test split at the patient level to prevent data leakage and ensure robust model evaluation. We also used a multi-center dataset to mitigate potential overfitting issues and improve the generalization of the model across different clinical settings. We recognize the potential benefits of data augmentation and plan to apply it in future studies to further improve the performance and robustness of our model. Thank you for raising this important point, which has helped us to reflect on the scope of our methodology.

2-4. Class Imbalance: How did the authors address class imbalance in the dataset?
**Response**

Thank you for raising the important issue of class imbalance in your dataset. In this study, we used Focal Loss for the RetinaNet model and ComputeLoss loss for YOLOv5 to address class imbalance so that the performance of the model is not biased towards the majority class. Specifically, we used [oversampling of the minority class, undersampling of the majority class, and a weighted loss function] during the training process. To prioritize the minority class, we applied a class weighted loss function to ensure that the model considered both classes equally during training. We also used evaluation metrics such as precision and sensitivity to ensure balanced model performance across all classes. These measures helped to mitigate the impact of class imbalance and improve the robustness of our results. Thank you for highlighting this aspect.

3. Model Selection
3-1. What motivated the selection of YOLOv5 and RetinaNet for this study? Were alternative models considered?
**Response**

Thank you for your question about the choice of YOLOv5 and RetinaNet for this study. These models were chosen because they have complementary strengths in object detection tasks, especially in the medical imaging context. YOLOv5 was chosen because of its high inference speed and efficiency, which makes it suitable for real-time clinical applications such as endoscopy. The architecture is well optimized to balance detection accuracy and computational complexity, which is critical in scenarios where rapid decision making is required. RetinaNet, on the other hand, was chosen because it is strong at detecting small and subtle lesions using a loss-of-focus function, which effectively solves the class imbalance problem and improves detection in challenging datasets like ours. Alternative models such as Faster R-CNN and SSD were considered, but Faster R-CNN was deemed unsuitable due to its relatively slow inference speed, which could hinder real-time application. SSD, while fast, performed poorly in preliminary tests on our dataset, particularly in detecting small lesions. Combining YOLOv5 and RetinaNet leverages the strengths of both models to achieve both speed and accuracy in our application. Thank you for clarifying these points and for your help with the model selection process.

3-2. Please provide details of any modifications made to the baseline architectures of YOLOv5 and RetinaNet.
**Response**

Thank you for your question regarding modifications to the baseline architecture of YOLOv5 and RetinaNet. In this study, we utilized YOLOv5l, one of the five model sizes available in the YOLOv5 suite, and applied certain modifications to optimize the performance of esophageal lesion detection. The prediction and training conditions were set to 200 epochs with a batch size of 16, image size of 640×640, and a learning rate of 1e-3 using the Adam optimizer. To calculate the final loss, we used the ComputeLoss function, which incorporates class loss, objectivity loss, and bounding box loss. We also used an early stopping algorithm to mitigate overfitting. For RetinaNet, the training conditions included batch size 1, image size 640×640, and learning rate 1e-5. To address the class imbalance inherent in the dataset, we used the Focal Loss function, a core feature of RetinaNet. We also modified the activation function by using the ReLU function in the backbone layer and the Sigmoid function in the final layer to improve classification performance. Similar to YOLOv5, we applied an early stopping algorithm to prevent overfitting during training. These adjustments were specifically designed to optimize the model for the unique characteristics of the dataset to ensure robust performance in esophageal lesion detection. We thank the reviewers for their comments, which allowed us to clarify these details in the revised manuscript.

4. Performance Evaluation
4-1. Comparison with State-of-the-Art: The results appear promising; however, the authors are encouraged to provide a direct comparison with state-of-the-art methods. While datasets may differ, comparisons with studies using esophageal datasets (e.g. leveraging WLI or NBI) would provide additional context. A summary table would be helpful for this purpose.

**Response**

Thank you for your valuable comments on the comparison with state-of-the-art methods. We agree that a direct comparison with studies utilizing esophageal datasets, particularly those utilizing WLI or NBI, would provide additional context and further strengthen the manuscript. However, due to the scope and focus of the current study, we did not include such a comparison in this work. Our primary goal was to validate the performance of the proposed method using the dataset collected for this study. We recognize the importance of benchmarking our results against state-of-the-art methods and plan to perform a comprehensive comparative analysis in future work. This will include exploring publicly available esophageal datasets and incorporating direct performance comparisons with existing studies to better contextualize our results. We sincerely appreciate the reviewer's suggestions, which highlight important directions for improving the impact and scope of our work in subsequent studies.

4-2. Confusion Matrix: To better visualize the model's performance, confusion matrices should be included, detailing metrics such as TP/FP/TN/FN. This will provide clearer insights into class-wise behavior.

**Response**
In response to your valuable suggestion, we have updated Tables 2 and 3 in the manuscript to include confusion matrices for both YOLOv5 and RetinaNet models in the Results section. These additions provide a clearer visualization of the models' performance and offer more detailed insights into class-wise behavior. We sincerely appreciate your thoughtful comment, which has helped us enhance the comprehensiveness of the paper.

Modified and supplemented contents – Page 12, Table 2, Page 14, Table 3

5. Considering PLOS ONE’s specific guidelines on code sharing ‘<https://journals.plos.org/plosone/s/materials-and-software-sharing#loc-sharing-code>’ for submissions involving author-generated code, I would encourage the authors to share their code in a way that supports the findings in the manuscript.

**Response**

We appreciate you raising the importance of code sharing in line with PLOS ONE's guidelines. In this study, we utilized the publicly available codebases of YOLOv5 and RetinaNet, which formed the basis of our work. These implementations can be found in the following GitHub repositories: YOLOv5 (https://github.com/ultralytics/yolov5) and RetinaNet (https://github.com/fizyr/keras-retinanet). Both repositories are publicly accessible and adhere to established best practices for reproducibility. While our work focused on adapting, fine-tuning, and optimizing these existing frameworks for esophageal lesion detection, we provided a detailed description of all modifications, hyperparameters, and experimental settings in the manuscript to ensure reproducibility. By referencing these publicly available repositories, we aim to maintain transparency and support reproducibility of our findings. We appreciate the reviewers' comments and are committed to maintaining the highest level of transparency and openness in our research practices.
